# Genetic deletion of mGlu2 metabotropic glutamate receptors improves the short-term outcome of cerebral transient focal ischemia

**DOI:** 10.1186/s13041-017-0319-6

**Published:** 2017-08-18

**Authors:** Federica Mastroiacovo, Slavianka Moyanova, Milena Cannella, Anderson Gaglione, Remy Verhaeghe, Giovanna Bozza, Michele Madonna, Marta Motolese, Anna Traficante, Barbara Riozzi, Valeria Bruno, Giuseppe Battaglia, David Lodge, Ferdinando Nicoletti

**Affiliations:** 1 IRCCS Neuromed, 86077 Pozzilli, Italy; 2 Department of Physiology and Pharmacology, Sapienza University of Rome, Piazzale Aldo Moro, 5, 00185 Rome, Italy; 3 School of Physiology, Pharmacology and Neuroscience, University of Bristol, Bristol, BS8 1TD UK

**Keywords:** Focal ischemia, mGlu2 receptor, Genetic deletion, Neuroprotection, Neurological score

## Abstract

We have recently shown that pharmacological blockade of mGlu2 metabotropic glutamate receptors protects vulnerable neurons in the 4-vessel occlusion model of transient global ischemia, whereas receptor activation amplifies neuronal death. This raised the possibility that endogenous activation of mGlu2 receptors contributes to the pathophysiology of ischemic neuronal damage. Here, we examined this possibility using two models of transient focal ischemia: (i) the monofilament model of middle cerebral artery occlusion (MCAO) in mice, and (ii) the model based on intracerebral infusion of endothelin-1 (Et-1) in rats. Following transient MCAO, mGlu2 receptor knockout mice showed a significant reduction in infarct volume and an improved short-term behavioural outcome, as assessed by a neurological disability scale and the “grip test”. Following Et-1 infusion, *Grm2* gene mutated Hannover Wistar rats lacking mGlu2 receptors did not show changes in the overall infarct volume as compared to their wild-type counterparts, although they showed a reduced infarct area in the agranular insular cortex. Interestingly, however, mGlu2 receptor-deficient rats performed better than wild-type rats in the adhesive tape test, in which these rats did not show the laterality preference typically observed after focal ischemia. These findings support the hypothesis that activation of mGlu2 receptors is detrimental in the post-ischemic phase, and support the use of mGlu2 receptor antagonists in the experimental treatment of brain ischemia.

## Introduction

Metabotropic glutamate (mGlu) receptors have been implicated in mechanisms of neurodegeneration/neuroprotection, and are promising drug targets for the treatment of acute and chronic neurodegenerative disorders [1]. It was believed for many years that group-II mGlu receptor agonists could produce neuroprotection by activating both mGlu2 and mGlu3 receptors [1], until it was shown in mouse primary cortical cultures that the protective activity of the mGlu2/3 receptor agonist, LY379268, against excitotoxic neuronal death was largely mediated by the activation of glial mGlu3 receptors [2]. The unexpected findings that cultured neurons lacking mGlu2 receptors were more resistant to excitotoxic death and that systemic administration of LY379268 was protective against 1-methyl-4-phenyl-1,2,3,6-tetrahydropyridine neurotoxicity only in mGlu2 receptor knockout mice [2] raised the possibility that mGlu2 receptors facilitated neuronal death. This hypothesis was supported by a further in vitro study in which neurotoxicity caused by β-amyloid peptide was amplified by a selective positive allosteric modulator (PAM) of mGlu2 receptors, which was inactive when neurons lacked mGlu2 receptors [3]. The unexpected neurotoxic activity of mGlu2 receptors contributes to explain the contrasting data obtained with orthosteric mGlu2/3 receptor agonists in in vitro models of excitotoxic neuronal death [4–6], and the suboptimal effect of these drugs in animal models of global and focal brain ischemia [7–10].

Using the 4-vessel occlusion model of transient global ischemia, we found that post-ischemic systemic treatment with a selective negative allosteric modulator (NAM) of mGlu2 receptors protected vulnerable hippocampal CA1 pyramidal neurons, whereas treatment with a PAM of mGlu2 receptors extended the damage to resistant CA3 pyramidal neurons [12]. The possibility that mGlu2 receptor NAMs may be used as protective agents in brain ischemia is particularly promising because mGlu2 receptor NAMs are currently under preclinical and clinical development for the treatment of major depression, and show cognitive enhancing properties associated with a good profile of safety and tolerability [13]. However, transient global ischemia in rodents represents a model of brain hypoperfusion associated with cardiac arrest or hypotensive shock, but has no translational value for drug development in the treatment of stroke. It is therefore necessary to extend the study of mGlu2 receptors to models of transient focal ischemia that more closely mimic thromboembolic stroke in humans, such as the models of transient middle cerebral artery occlusion (MCAO) induced by filament insertion in mice [14] and intracerebral endothelin-1 (Et-1) infusion in rats [15]. We used these two models respectively in mGlu2 receptor knockout mice (mGlu2^−/−^) and in Hannover Wistar rats (Han Wistar rats) [16, 17] lacking mGlu2 receptors to examine the influence of mGlu2 receptors on the short-term histological and behavioural outcome of transient focal ischemia.

## Results

### Infarct volume and improved behavioral outcome in mGlu2 receptor knockout mice subjected to transient MCAO

**Fig. 1:**
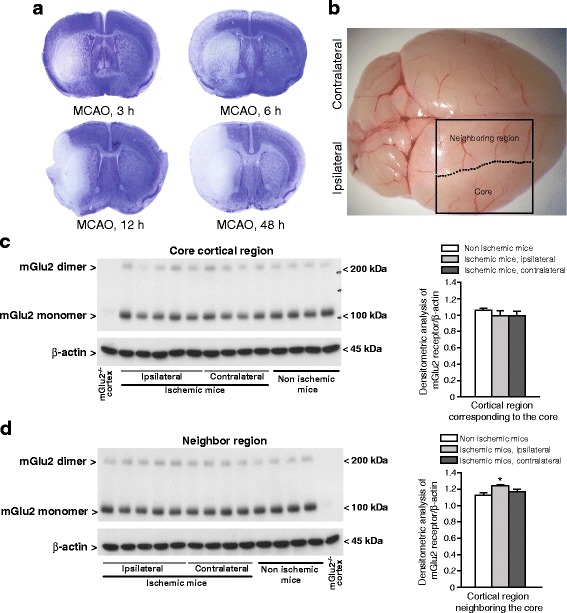
mGlu2 Receptor protein levels in the cerebral cortex of mGlu2^+/+^ mice subjected to transient MCAO. Representative images of Nissl staining in coronal sections of mice at 3, 6, 12 and 48 h after monofilament-induced transient MCAO are shown in (**a**). The boundaries of dissection of the core region and the neighboring cortical region used for Western blot analysis are shown in (**b**). Immunoblot analysis of mGlu2 receptors in the cortical region corresponding to the core and in the cortical regions neighboring the core (and the corresponding regions of the contralateral hemispheres and of non-ischemic mice) is shown in (**c**) and (**d**), respectively. Densitometric data were obtained as the sum of the two bands corresponding to monomers and dimers. Data are means + S.E.M. of 4–5 mice per group. *p < 0.05 as compared to all other groups (One-way ANOVA + Fisher’s PLSD; F_2,10_ = 9.26)

Transient MCAO in mGlu2^+/+^ mice caused a rapid development of ischemic infarct, which involved the striatum and a large proportion of the surrounding cerebral cortex. The ischemic infarct was detectable as early as 3 h after reperfusion, and reached its maximal size at 24–48 h after reperfusion (Fig. 1a). To examine whether MCAO could induce adaptive changes in the expression of mGlu2 receptors, we measured mGlu2 receptor protein levels by Western blot analysis in the cerebral cortex at 6 h after reperfusion. Measurements were performed in the core of the infarct area, in the neighboring cortical area, in the corresponding regions of the contralateral intact hemisphere, and in the corresponding regions of sham operated mice (see Fig. 1b). Western blot analysis showed a band at about 100 kDa corresponding to mGlu2 receptor monomers, and a higher molecular size band, corresponding to receptor dimers. The identity of the two bands was confirmed using reference tissue from mGlu2^−/−^ mice (see immunoblot in Fig. 1c, d). mGlu2 receptor protein levels were not altered in the core region of the ischemic cerebral cortex as compared to the contralateral cortex or to the cortex of non-ischemic mice (Fig. 1c). In contrast, a slight but significant increase in mGlu2 receptor protein levels was seen in the cortical region neighboring the ischemic core as compared to the contralateral region and to the corresponding region of non-ischemic mice (Fig. 1d).

**Fig. 2:**
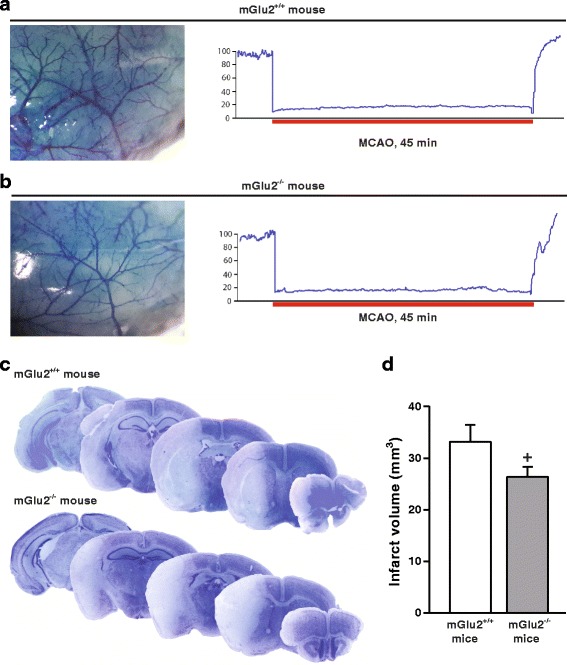
Reduced infarct volume in mGlu2^−/−^ mice subjected to transient MCAO. Evans blue perfusion showing the absence of anatomical abnormalities of the MCA mGlu2^−/−^ with respect to mGlu2^+/+^ mice (**a**, **b**). No difference in the reduction of cerebral blood flow in response to MCAO was found among mGlu2^+/+^ and mGlu2^−/−^ mice. Nissl staining of mGlu2^+/+^ and mGlu2^−/−^ mouse brains after transient MCAO. Mice were killed 48 h after reperfusion (**c**). Values of infarct volume (**d**) are means ± S.E.M. (*n* = 9 per group). ^**+**^ *p* < 0.05 (Student’s t-test; t_16_ = 1.75)

We next examined whether the lack of mGlu2 receptors could affect the extent of the ischemic lesion in mice subjected to transient MCAO induced by filament insertion into the external carotid artery. Macroscopic analysis of cerebrovascular anatomy did not show differences between mGlu2^−/−^ mice and their wild-type counterparts (mGlu2^+/+^). In addition, MCAO reduced regional cerebral blood flow (rCBF) to a similar extent in the two genotypes (Fig. 2a, b). The volume of ischemic infarct, evaluated by Nissl staining at 48 h after reperfusion, was significantly smaller in mGlu2^−/−^ mice, as compared to mGlu2^+/+^ mice (Fig. 2c, d).

**Fig. 3:**
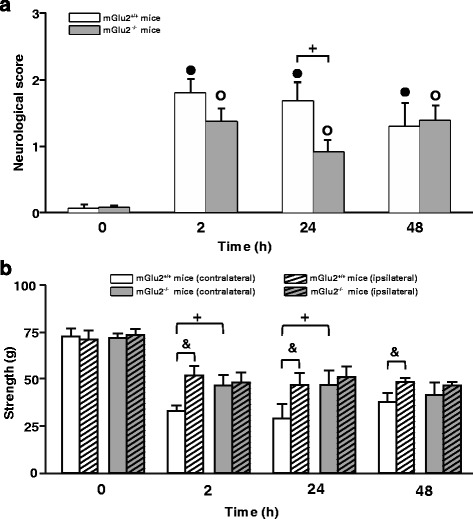
Improved short-term behavioral outcome in mGlu2^−/−^ mice subjected to transient MCAO. Neurological deficit in mGlu2^+/+^ and mGlu2^−/−^ mice subjected to transient MCAO (**a**). *Bars* are means + S.E.M. Friedman nonparametric ANOVA revealed significant effect of Time for mGlu2^+/+^ mice [Chi Sqr._(*N*=7, df=4)_ = 23.46, *p* = 0.0001] and for mGlu2^−/−^ mice [Chi Sqr._(*N*=9, df=4)_ = 31.62, *p* = 0.000001]; (**●**) and (O) are Wilcoxon matched pairs comparisons between T_0_ (before ischemia) and 2, 24 and 48 h for mGlu2^+/+^ mice (*n* = 9) and mGlu2^−/−^ mice (*n* = 11), respectively; ^**+**^ Kruskal-Wallis ANOVA and post-hoc Kruskal-Wallis test by ranks for difference between mGlu2^+/+^ and mGlu2^−/−^ mice. Grip strength of ipsilateral and contralateral FL in mGlu2^+/+^ and mGlu2^−/−^ mice subjected to transient MCAO (**b**). MCAO resulted into a significant reduction in muscular strength in both, ipsilateral and contralateral FLs, after ischemia compared to T_0_ in mGlu2^+/+^ (*n* = 7) and mGlu2^−/−^ mice (*n* = 8) (*p* < 0.05, Dunnett’s t test). Three-way ANOVA for repeated measures (first factor GROUP with two levels: mGlu2^+/+^ and mGlu2^−/−^ mice, second repeated measure factor SIDE with two levels: ipsilateral FL and contralateral FL, and third repeated measure factor TIME with four levels: T_0_, 2, 24, and 48 h) revealed significant effects of GROUP (F_1,9_ = 7.11, *p* = 0.026), SIDE (F_1,9_ = 6.69, *p* = 0.029), TIME (F_3,27_ = 21.16, *p* = 0.0000001), interactions SIDE x TIME (F_3,27_ = 3.83, *p* = 0.021) and GROUP x SIDE x TIME (F_3,27_ = 4.59, *p* = 0.01). ^&^ Comparisons between ipsilateral and contralateral FL in mGlu2^+/+^ mice (Fisher LSD test). ^**+**^ Comparisons between mGlu2^+/+^ and mGlu2^−/−^ mice for the contralateral FL (Fisher LSD test)

In mGlu2^+/+^ mice, MCAO induced a neurological deficit with a score of about 2 (weakness and circling behavior towards the contralateral site) at 2 and 24 h after reperfusion. The deficit was attenuated at 48 h. The neurological deficit was significantly reduced in mGlu2^−/−^ at 24 h (Fig. 3a), but not at 48 h after ischemia.

The most striking difference between the two genotypes was observed when muscular strength of the left and right forelimbs was measured by the grip test. mGlu2^+/+^ mice showed a marked asymmetry in muscular strength, which was reduced in the contralateral forepaw at 2, 24, and 48 h after reperfusion. No asymmetry was found in mGlu2^−/−^ mice at any time point (Fig. 3b). The loss of muscular strength was significantly greater in mGlu2^+/+^ mice than in mGlu2^−/−^ mice at 2 and 24 h after reperfusion (Fig. 3b).

**Fig. 4:**
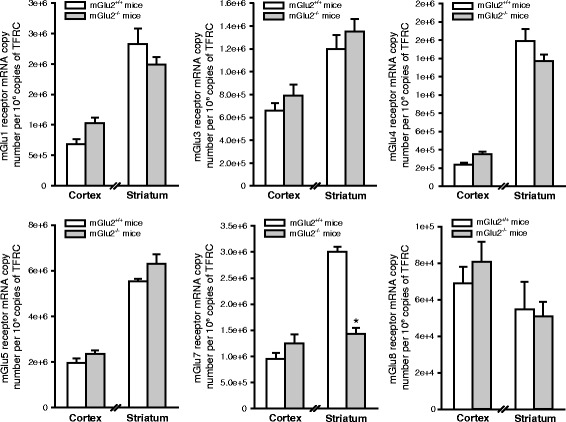
Analysis of mGlu receptor subtypes in the cerebral cortex and striatum of mGlu2^+/+^ and mGlu2^−/−^ mice. Quantitative PCR analysis of mGlu1, mGlu3, mGlu4, mGlu5, mGlu7 and mGlu8 receptors mRNA levels in mGlu2^+/+^ and mGlu2^−/−^ mice. mRNA values, expressed as copy number, were normalized to TFRC (transferrin receptor protein-1) and are means + S.E.M. of 4–8 mice per group. * *p* < 0.05 (Student’s t test; t_10_ = 4.59)

To exclude the possibility that mGlu2^−/−^ mice could be protected against ischemic damage because of compensatory changes in other mGlu receptor subtypes, we measured the transcripts of mGlu1, mGlu3, mGlu4, mGlu5, mGlu7, and mGlu8 receptors in the cerebral cortex and striatum of mGlu2^−/−^ mice and their wild-type littermates. We found no changes in the transcripts of the various mGlu receptor subtypes with the exception of the transcript of mGlu7 receptors, which was significantly reduced in the striatum of mGlu2^−/−^ mice (Fig. 4).

### Short-term outcome of transient focal ischemia in wild-type and *Grm2* mutant Han Wistar rats

**Fig. 5:**
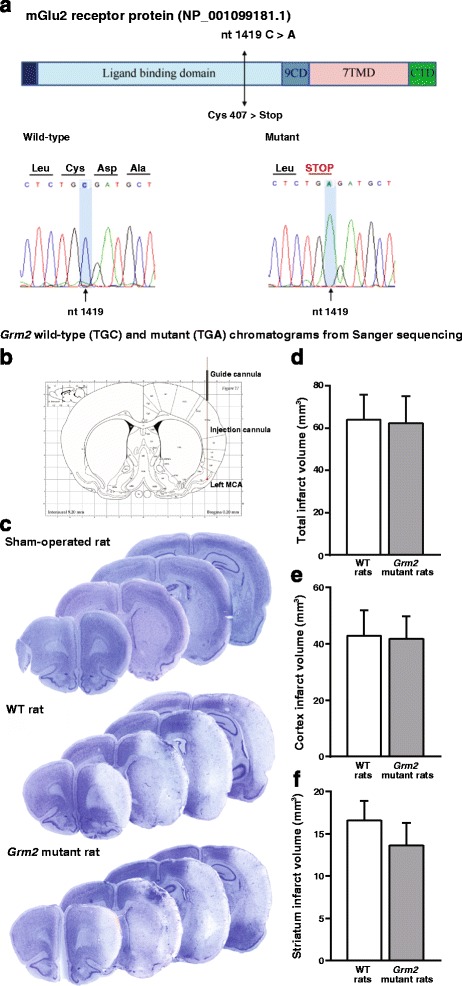
No difference in the infarct volume between WT and *Grm2* mutant rats subjected to MCAO. WT and *Grm2* mutant chromatograms from Sanger sequencing (**a**); scheme design of a coronal brain section at bregma level + 0.2 mm [30] (**b**); representative images of brain Nissl staining in non-ischemic (sham-operated rat), WT and *Grm2* mutant Han Wistar rats subjected to transient MCAO (**c**); total infarct volume and infarct volumes in the cerebral cortex and striatum are shown in (**d**), (**e**), and (**f**), respectively. *Bars* represent means + S.E.M. of 8 rats per group

**Fig. 6:**
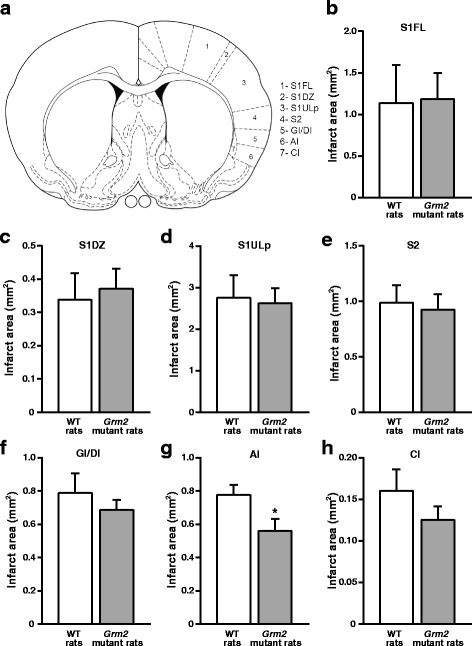
Reduced infarct area in the agranular insular cortex of *Grm2* mutant rats subjected to MCAO. Infarct areas of cortical sub-regions examined in one microscopic section (**a**) corresponding to the level of Et-1 infusion **(**AP **=** + 0.2 mm) are shown in (**b**-**h**). *Bars* are means + S.E.M. of 8 rats per group. * *p* < 0.05 (Student’s t test). S1FL, S1DZ, and S1ULP = forelimb region, dysgranular region, and upper lip region of the primary somatosensory cortex, respectively; S2 = secondary somatosensory cortex; GI/DI, AI = granular/dysgranular, and agranular insular cortex, respectively; Cl = claustrum

We could identify Han Wistar rats carrying a single point mutation of exon 3 of the *Grm2* gene, which results into a stop codon at Cys407 and the lack of functional mGlu2 receptors [17] (Fig. 5a). Wild-type (WT) and *Grm2* mutant Han Wistar rats were subjected to transient focal ischemia induced by unilateral injection of Et-1 near the MCA of the left hemisphere (Fig. 5b). MCAO caused a large infarct volume detected after 72 h, which did not differ between WT and *Grm2* mutant rats (Fig. 5c, d). There was no difference in the infarct volume in the cerebral cortex and striatum between ischemic WT and *Grm2* mutant Han Wistar rats, although a trend to a reduction in *Grm2* mutant Han Wistar rats was observed (Fig. 5e, f). We also measured infarct area in different cortical sub-regions in a single section at +0.2 mm from bregma, corresponding to the site of Et-1 injection (Fig. 6a). No significant differences in the infarct area between WT and *Grm2* mutant rats were found in the forelimb region (S1FL), dysgranular region (S1DZ), and upper lip region (S1ULp) of the primary somatosensory cortex and in the secondary somatosensory cortex (S2) (Fig. 6b, e). *Grm2* mutant rats showed a significant reduction in the infarct area in the agranular insular cortex (AI) (Fig. 6g), and a trend to a reduction in the granular and dysgranular insular cortex (GI/DI) and claustrum (Cl) (Fig. 6f, h).

**Fig. 7:**
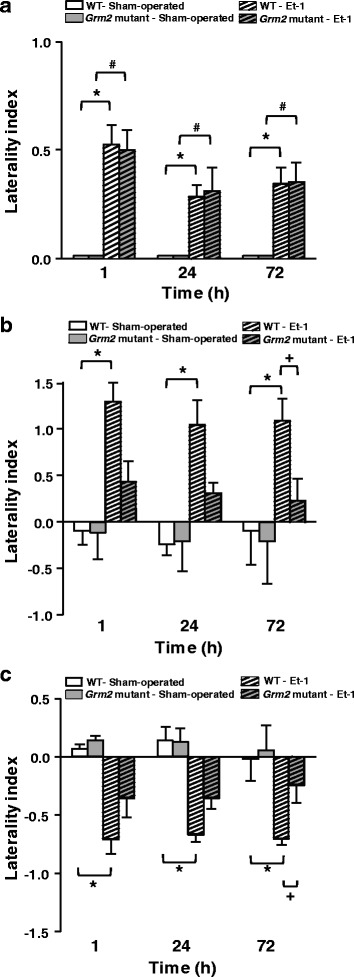
Improved motor asymmetry in *Grm2* mutant Han Wistar rats subjected to transient MCAO. Asymmetry (expressed by laterality index, normalized to pre-ischemia values) in the PHR test is shown in (**a**). Kruskal-Wallis nonparametric ANOVA and Mann-Whitney comparisons between Et-1-infused and sham-operated rats: (*) for WT rats and (^**#**^) *Grm2* mutant rats, respectively; Asymmetry in the preference and latency of removing the adhesive tapes in the AT test is shown in (**b**) and (**c**), respectively. Three-way GLM rANOVA (first factor ISCHEMIA with two levels: Et-1 and sham-operated, second factor GROUP with two levels: WT and *Grm2* mutant rats, and third factor TIME with three levels: 1, 24 and 72 h) revealed a significant effect of factor ISCHEMIA for all rats, as follows: Preference (F_(1,15)_ = 12.84, *p* = 0.003) and Latency (F_(1,15)_ = 28.31, *p* = 0.00009). For ischemic rats (infused with Et-1), two-way rANOVA with factors GROUP (WT and *Grm2* mutant rats) and TIME (1, 24 and 72 h) showed significant effect of the factor GROUP for both AT parameters, as follows: Preference (F_(1,10)_ = 10.84, *p* = 0.008) and Latency (F_(1,10)_ = 10.83, *p* = 0.008). * Fisher LSD comparisons between Et-1 and sham-operated WT rats and ^**+**^ between WT- Et-1 and *Grm2* mutant-Et-1 rats. Number of rats: WT-sham-operated (*n* = 3); *Grm2* mutant Han Wistar rats-sham-operated (*n* = 4); WT-Et-1 (*n* = 7); *Grm2* mutant Han Wistar rats-Et-1 (*n* = 8). Significance was set at *p* < 0.05

Behavioral assessment with a neurologic examination grading system that included a postural hang reflex test (PHR) showed a moderate to severe neurologic deficit in the right side of the body in both genotypes (Fig. 7a). Statistical analysis of asymmetry expressed by the laterality index revealed a significant effect of ischemia at all time sessions, as compared to pre-ischemic values (T_0_), in both WT and *Grm2* mutant rats (Fig. 7a).

A difference between the two strains of rats emerged from the analysis of preference and latency of removing the adhesive tapes in the adhesive tape test (AT) test, which is an active test of sensorimotor integration [18]. We found a large and significant increase in asymmetry in both parameters of the AT test (preference and latency) in WT rats at all times after Et-1 infusion (1, 24, and 72 h) (Fig. 7b, c). This was indicative of a severe defect in an active behavioral performance that requires sensorimotor integration [18]. Ischemia-induced asymmetry was much less severe in *Grm2* mutant rats than in the WT rats. While the laterality index in the ischemic WT rats differed significantly from that in sham-operated WT rats, the laterality index in sham-operated and Et-1 infused *Grm2* mutant rats was not significant different at all time points (Fig. 7b, c). There was a significant difference in laterality index between ischemic WT and *Grm2* mutant rats at 72 h after Et-1 infusion (Fig. 7b, c).

## Discussion

Neuroprotection remains an unmet need in the treatment of ischemic stroke. Disappointing data were obtained in clinical studies with ionotropic glutamate receptor antagonists in stroke owing to the strong impact of these drugs on fast excitatory synaptic transmission in the CNS, and the opposite role played by synaptic and extrasynaptic N-methyl-D-aspartate (NMDA) receptors in neurodegeneration/neuroprotection [1–19]. In addition, NMDA receptor antagonists impair mechanisms of activity-dependent synaptic plasticity and may cause severe adverse effects, such as psychotomimetic effects and intrinsic neurotoxicity [1]. mGlu receptor ligands *modulate* synaptic transmission, and are therefore considered as more valuable candidate targets for neuroprotective drugs. The mGlu receptor ligands developed so far for the treatment of CNS disorders have shown a good profile of safety and tolerability, which may reflect the *modulatory* role of mGlu receptors in synaptic transmission and activity-dependent synaptic plasticity.

Our interest in mGlu2 receptors as targets for neuroprotective drugs was raised by in vitro studies showing that the neuroprotective activity of mGlu2/3 receptor agonists against NMDA or β-amyloid toxicity was largely mediated by mGlu3 receptors, and that activation of mGlu2 receptors was instead neurotoxic [2, 3]. These findings laid the groundwork for an in vivo study in which we examined mGlu2 receptor expression in vulnerable and non-vulnerable hippocampal subregions and the effects of selective mGlu2 receptor ligands in the 4-vessel occlusion model of transient global ischemia. We found that mGlu2 receptor mRNA levels were lower in the resistant hippocampal CA3 region than in the vulnerable CA1 region, and that transient global ischemia selectively down-regulated mGlu2 receptors in the CA1 region [12]. In addition, we found that systemic post-ischemic treatment with a selective mGlu2 receptor NAM protected CA1 neurons against ischemic damage, whereas treatment with a selective mGlu2 receptor PAM extended the damage to CA3 neurons [12].

We have now studied the role of mGlu2 receptors in ischemic damage and the resulting behavioral impairment using two models of transient focal ischemia: (i) the monofilament model in mice; and, (ii) the Et-1 model in rats. In mice, transient MCAO did not induce early changes in the expression of mGlu2 receptors in a cortical area corresponding to the ischemic core, whereas it caused a significant increase in mGlu2 receptor protein levels in the neighboring cortical area that likely includes part of the penumbra region. This increase was small and its significance in the pathophysiology of ischemic damage is uncertain. However, these data further suggest that the ischemic insult causes adaptive changes in the expression of mGlu2 receptors.

Infarct size was significantly reduced in ischemic mGlu2^−/−^ mice as compared to their WT counterparts, but not in *Grm2* mutant rats, although the extent of ischemic damage was similar in mice and rats following transient MCAO. The easiest explanation for this apparent discrepancy is that it is not the lack of mGlu2 receptors that protects mGlu2^−/−^ mice against damage caused by transient focal ischemia. Compensatory changes in the expression of other receptors or membrane transporters might render these mice less vulnerable to ischemic damage. It was logical to examine whether expression of other mGlu receptor subtypes was abnormal in the cerebral cortex and striatum of mGlu2^−/−^ mice. We found no changes in the transcripts of mGlu receptor subtypes that have been implicated in mechanisms of neurodegeneration/neuroprotection, such as mGlu1, mGlu3, mGlu4, and mGlu5 receptors [1, 20, 21]. In contrast, the transcript of the mGlu7 receptors was substantially reduced in the striatum - but not in the cerebral cortex - of mGlu2^−/−^ mice. We do not believe that this reduction contributes to reduce vulnerability of mGlu2^−/−^ mice to ischemic damage because the mGlu7 receptor negatively modulates the activity of NMDA receptors [22], and, therefore, its activation is potentially neuroprotective. No need to say that possible alterations in other neurotransmitter receptors, membrane transporters or intracellular proteins might contribute to the reduced vulnerability of mGlu2^−/−^ mice to ischemic damage. This hypothesis warrants further investigation.

Interestingly, the absence of mGlu2 receptors improved the behavioral outcome of focal ischemia in *both* mice and rats. In mGlu2^−/−^ mice neurological disability was significantly attenuated at 24 h, but not at 48 h after ischemia, and the defect in the grip strength in the contralateral forearms was prevented in these mice at all time points after ischemia. *Grm2* mutant rats lacking mGlu2 receptors showed a substantial improvement in their performance in the AT test. This is an active test of sensorimotor integration involving the somatosensory cortex, anteromedial cortex, and striatum [23, 24]. The AT test, also called sensorimotor asymmetry test, models the tactile neglect in patients with frontal and parietal cortical lesions [25, 26]. We were surprised to find that ischemic *Grm2* mutant rats showed less asymmetry in the tape test as compared to WT rats, in spite of the similar extent of ischemic infarct in the two genotypes. Ischemic *Grm2* mutant rats showed a significant reduction of the infarct area in the dorsal portion of the AI, and a trend to a reduction in the granular and GI/DI and Cl. The Cl is involved in the processing of sensorimotor and visuomotor information [27]. The dysgranular portion of the insular cortex receives projections from the secondary somatosensory area and, therefore, is also involved in somatosensory processing [28]. In contrast, the agranular portion of the insular cortex, which is part of the pain matrix, is involved in nociceptive processing [29], and may play a role in decision-making behavior under risk [30]. Of note, activation of the insular cortex is associated with orofacial movements [31], which are involved in sensorimotor integration during performance of adhesive tape removal. It is possible that in the post-ischemic period the application of a tape in the forepaw is processed as an aversive event and that activation of the AI is required for the decision to rapidly remove the tape. If so, the reduced infarct area in the AI might contribute to the better performance of mGlu2^−/−^ mice in the AT test. However, this is not fully convincing, and the combination of mouse and rat data suggest that, at least in models of focal ischemia, endogenous activation of mGlu2 receptors may not be critical for the development of ischemic neuronal damage, but becomes detrimental for functional recovery at short intervals after reperfusion. The use of selective mGlu2 receptor ligands will be a necessary step for an in-depth investigation of the role played by mGlu2 receptors in models of transient focal ischemia.

## Conclusions

Our data suggest that endogenous activation of mGlu2 receptors is detrimental for the short-term outcome of transient focal ischemia, and support the hypothesis that pharmacological blockade of mGlu2 receptors is a valuable strategy for the treatment of brain ischemia. It is possible that endogenous activation of mGlu2 receptors contributes to dysfunction of synaptic plasticity and network activity underlying the imbalance between the two hemispheres in the early post ischemic phase, and that mGlu2 receptor antagonists correct this dysfunction thereby facilitating functional recovery. This is consistent with the “therapeutic” effect of mGlu2 receptor antagonists in experimental animal models of cognitive dysfunction associated with major depression [13].

The reduced infarct volume observed in mGlu2^−/−^ mice subjected to transient focal ischemia leaves open the possibility that endogenous activation of mGlu2 receptors plays a permissive role in ischemic neuronal damage, although data obtained in *Grm2* mutant rats are not consistent with this hypothesis. Further studies are needed to better understand the precise role played by mGlu2 receptors in the pathophysiology of ischemic neuronal death.

## Methods

### Animals

mGlu2^−/−^ mice on a C57Bl/6 genetic background were kindly provided by Prof. Shigetada Nakanishi (Osaka, Japan) and the colony was generated by homozygous breeding. Han Wistar rats were purchased by Jackson Laboratories (Bar Harbor, ME) and genotyped for the presence of a stop codon mutation in the *Grm2* gene (see below). All animals were housed under standard conditions with food and water ad libitum and a 12:12 h light-dark cycle. Studies were performed in agreement with the national and international guidelines and regulations on animal care and use, and were approved by the Neuromed Institutional Animal Care and Use Committee. All efforts were made to minimize animal suffering and to reduce the number of animals used.

### Induction of transient MCAO in mice

#### Surgery, stroke induction, inclusion criteria

For measurements of mGlu2 receptor protein levels in the cerebral cortex at 6 h following ischemia, we used 4 non-ischemic and 5 ischemic C57Bl mice (20–25 g, b.w.). For the study of focal ischemia in mice lacking mGlu2 receptors we used 15 male mGlu2^−/−^ mice and 11 age-matched male mGlu2^+/+^ mice (20–25 g, b.w.). Transient MCAO occlusion was induced as described by Nygren and Wieloch [14]. Briefly, anesthesia was induced by inhalation of 2.5% isoflurane in N_2_O/O_2_ (70:30) and maintained at 2% by a face mask during the initial phase of surgery. Body temperature was maintained at 37 °C using a heating pad and controlled by a device connected with a temperature probe inserted into the rectum. rCBF was monitored by a flexible optical fiber connected to a laser Doppler (PeriFlux System 5000; Perimed, Jarfalla, Sweden) mounted on the skull in correspondence of the MCA of the right hemisphere. A silicon-coated filament (6–0 MCAO suture, Doccol Corporation, Sharon MA) was introduced into the internal carotid artery through an incision in the external carotid artery. The filament was advanced until it blocked the origin of the right MCA. Filament placement was confirmed by a reduction in laser Doppler flow and then, the isoflurane concentration was decreased to 1.5% during the MCA occlusion (45 min). We have included into analysis only mice with adequate occlusion, as determined by (1) rCBF reduction by at least 70% immediately after filament placement; (2) sustained reduction of rCBF for 45 min during occlusion; and, (3) complete recovery of rCBF within 5 min after the filament was removed. Therefore, 9 mGlu2^+/+^ and 9 mGlu2^−/−^ mice were included in statistical analysis for evaluation of the infarct volume. Nine mGlu2^+/+^ and 11 mGlu2^−/−^ mice were included in statistical analysis for evaluation of neurological deficits. Two mGlu2^−/−^ mice died before performing the last behavioural test and therefore were not included in the infarct volume analysis. Seven mGlu2^+/+^ and 8 mGlu2^−/−^ mice were used for statistical analysis of the grip strength test. After surgery, mice were injected with 0.5 ml of 5% glucose subcutaneously, placed into an incubator (Compact incubator, Thermo Scientific, AHSI, Bernareggio (MI), Italy) at 37C° for 2 h, and then returned back to their home cages. Animals were killed 48 h after MCAO and their brains processed for histologic analysis.

### Western blot analysis of mGlu2 receptors following transient focal ischemia

Ischemic mice were killed by decapitation 6 h following reperfusion. A cortical area corresponding to the ischemic core (identified by the presence of a white boundary from the surrounding tissue) and the neighboring cortical area (see Fig. 1b) were dissected. The corresponding regions of the contralateral hemispheres and the corresponding regions of the cerebral cortex of sham-operated mice were also dissected and stored frozen at −80 °C. Samples homogenized at 4 °C in in a solution containing Tris-HCl (pH 7.5), NaCl (50 mM), EDTA (5 mM), and an Ultra Cruz Protease Inhibitor cocktail. Ten μg of proteins from the supernatants were separated by 8% SDS polyacrilamide gel. Proteins were transferred on immuno-blot PVDF membranes (Trans-Blot Turbo Transfer Systems, Bio-Rad, Segrate, MI, Italy), which were incubated with a polyclonal anti-mGlu2 receptor antibody (Abcam, Cambridge, UK; 1:1000 in t-TBS) for 1 h at room temperature, and then incubated for 1 more h with an anti-mouse secondary antibody (Calbiochem, San Diego, CA; 1:7000 in t-TBS). For β-actin immunostaining, membranes were incubated with a mouse monoclonal antibody (Sigma-Aldrich, St Louis, MO; 1:50,000) in milk 5%, for 1 h at room temperature, and then incubated for 1 more h with an anti-mouse secondary antibody (Calbiochem; 1:7000 in t-TBS). Immunostaining was revealed by the enhanced ECL western blotting analysis system.

### Behavioral tests

#### Evaluation of neurological function

We assessed the neurological function 1 d before (T_0_) and at 2, 24, and 48 h after MCAO. Neurological function was scored by an investigator who was unaware of the genotype. We used the following grading system: 0 = no deficit; 1 = forelimb weakness; 2 = circling toward the affected, contralateral (left) side; 3 = partial paralysis of the affected side; 4 = no spontaneous motor activity [32].

#### Grip strength test

We assessed neuromuscular function by using the Grip strength meter (2 Biological Instruments, Besozzo, VA, Italy). The mouse voluntarily gripped a bar with either the healthy (ipsilateral) or the affected (contralateral) forelimb (FL) and pulled it backward. A mean of five trials was used for analysis. We assessed muscular strength one day before ischemia (T_0_) and then, at 2, 24, and 48 h after MCAO.

### Quantitative analysis of the transcripts of mGlu1, −3, −4, −5, −7, and −8 receptors in the cerebral cortex and corpus striatum of non-ischemic mGlu2^+/+^ and mGlu2^−/−^ mice

Mice were killed by decapitation and the brain was quickly removed; a cortical area corresponding to both dissected regions in Fig. 1b, and the striatum were dissected on ice and immediately frozen on liquid nitrogen, and stored at −80 °C. Total RNA was extracted using Trizol reagent (Thermo Fisher Scientific, Waltham, MA) according to manufacturer’s protocol. The RNA was then treated with DNAse (Qiagen, Hilden, Germany) and single strand cDNA was synthesized from 2 μg of total RNA using superscript III (Thermo Fisher Scientific) and random hexamers. Real-time PCR was performed as described previously [12]. The following primers were used: mGlu1 receptor, forward CATACGGAAAGGGGAAGTGA and reverse AAAAGGCGATGGCTATGATG; mGlu3 receptor, forward CAGCAAGCTCCCTCTTTTGT and *Rev.* GCTAAAAGAGCCCGTCACTG; mGlu4 receptor, forward CTCCAGCCGCACGCTTGACA and reverse GTAGGCCGAGTCCTGCCCGA; mGlu5 receptor, forward ACGAAGACCAACCGTATTGC and reverse AGACTTCTCGGATGCTTGGA; mGlu7 receptor forward GGTTTTCGTCAAGCCAGAGA and reverse ATCACTGAGTTCAGGAGCCG; mGlu8 receptor forward CGGAATCTGAACTTGCTCGG and reverse GGGGGAAGGCTTTAGGGATTT; and TFRC (transferrin receptor protein-1) forward CCAGTGTGGGAACAGGTCTT and reverse GCACCAACAGCTCCAAAGTC.

### DNA extraction, PCR amplification and sanger sequencing method for Han Wistar genotyping

DNA was extracted from the tail using Wizard genomic DNA purification kit (Promega Corporation, Madison, WI) according to manufacturer’s protocol. Fifty μg of DNA was used for PCR amplification with following primers forward: - 5′ GAACAGGAGTCAAAGATCATG 3′ and reverse: - 5′ CAGCACTATTACCGTCAAAC 3′. Thermal cycler conditions were as follows:10 min at 95 °C, 35 cycles of denaturation (30 s at 95 °C), annealing (30 s at 55°) and extension (45 s at 72 °C); the extension was continued at 72 °C for 7 min. Five μm of amplificated DNA was separated in a 2% agarose gel. All positive PCR products were purified by Minielute PCR purification kit (Qiagen) by standard procedures and 5 ng were sequenced with Big Dye Terminator v. 3.3 mix (Thermo Fisher Scientific) with forward primer by using the following thermal cycler conditions program: 30 cycles of denaturation (10 s at 95°) annealing (5 s at 54°) and extension (4 min 60°). Excess dye terminators were removed using 2.0 spin kit (Qiagen); samples were electrophoresed on an ABI Prism 310 genetic analyzer (Applied Biosystems, Foster City, CA) for genotype analysis.

### Et-1-induced focal ischemia in mutant Han Wistar rats

#### Surgery, stroke induction, inclusion criteria

Twenty-six rats (weight 310 ± 40 g) were used: 13 *Grm2* mutant Han Wistar and 13 WT. We used the Et-1 model of ischemia for the induction of transient focal ischemia. Rats were anaesthetized with an intramuscular injection of a mix of ketamine (60 mg/kg) and xylazine (12 mg/kg) dissolved into saline. A guide cannula was placed at the following stereotaxic coordinates: AP +0.2; ML +5.2 [33] avoiding to damage the *dura mater*. A stainless steel wire was insert into the guide cannula to plug it until the day of the Et-1 infusion. The guide cannula was secured to the skull with two supporting screws and dental cement. We infused Et-1 (200 pmol in 3 μl of saline) 15–16 d after the surgery (a 5 days-treatment with Baytril antibiotic, 0.2 ml/kg, s.c., 3 d of handling, 5 d of habituation to the experimental room and experimental cage and training in the AT test, and 3 d for control sessions before the Et-1 infusion). Et-1 was infused in non-anesthetized rats by inserting an injection cannula into the guide cannula. The tip of the injection cannula targeted the piriform cortex approximately 0.2 mm from the MCA origin in the left hemisphere. The same procedure was carried out in sham-operated rats, infused with vehicle. We assessed the ischemia-induced neurological deficits 10–15 min after Et-1 infusion using the following 7-point scale: 0 = normal; 1 = held to face the table edge (to avoid vibrissae and snout contacts with the table, the rat chin was supported upward), the rat failed to place its right FL on the table when we pushed the limb down with a soft bar (failed proprioceptive dorsal FL placing); 2 = held and slightly pushed to face the table edge from inside, the rat failed to hold its FL on the edge of the table and slipped off (failed proprioceptive ventral FL placing); 3 = suspended by the tail, the rat twisted the torso upward; 4 = suspended by the tail, the rat failed to extend the right FL down, and the limb was flexed and/or clenched; 5 = after being placed on the table, the rat turned its body to the right (spasmodic turning); 6 = the rat circled continuously to the right after being placed on the table. Ischemia was considered as severe with scores from 11 to 21, moderate, from 3 to 10; slight from 1 to 2. Twenty-two rats (10 WT and 12 *Grm2* Han Wistar mutant) satisfied these inclusion criteria.

### Behavioural tests

#### Neurological assessment and PHR test

We assessed spontaneous activity, symmetry in limb movement, forepaw outstretching, and resistance to lateral push and circling behavior [34]. In detail, we assessed: (i) flexion and/or clenching of the digits and/or full flexion of the wrist when the rat was suspended by the tail (PHR test); (ii) asymmetry in resistance to applied lateral gentle pressure from behind the shoulders in the left and right directions (the rat was placed on a flat surface). Ischemic rats showed less resistance of the right part of the body (contralateral to the ischemic hemisphere). The right FL became stiff during the push, while the left FL offered resistance; (iii) body twisting when the rat was suspended by the tail (PHR test); and, (iv) circling or inability to walk straight when the rat was placed on a flat surface. The following 4-score grading system was adopted: 0 = impairment in (i), (ii), (iii), and (iv) (severe motor deficit); 1 = impairment in (i), (ii), and (iii) (moderate motor deficit); 2 = impairment in (i) and (ii) (mild motor deficit); 3 = no deficit.

#### AT test

Two adhesive tapes (1 × 1 cm) were placed on the radial aspect of both FLs alternating the order of their application, i.e., right versus left. Both ATs were then pressed slightly and simultaneously and rats were placed immediately within a box and the time in seconds (precision of measuring 1.0 s) needed to remove the ATs (latency) and the order (preference) of FLs (left or right) for removing the first AT was recorded. Rats were pre-trained (five trials for five successive days) to obtain optimal level of performance (latency ≤28 s) and to achieve absence of asymmetry between right and left FL performance before stroke induction. The experimenter was blind to the rat group (Et-1, sham-operated, WT or *Grm2* mutant). A trial ended when three min had elapsed without removing of either of the two ATs. For the preference, the ipsilateral (Ipsi) and contralateral (Contra) values referred to mean percentage of trials (out of five for each pre- or post-Et-1 session) in which the rat removed the AT placed on the left or right FL, respectively. For the latency, the Ipsi and Contra values referred to mean time (for five trials in each pre- or post-Et-1 session) needed to remove the AT from the ipsilateral or contralateral FL, respectively.

### Asymmetry assessment in behavioural performance (AT and PHR tests)

The asymmetry in behavioural performance was assessed by means of the Laterality Index (LI) = (Ipsi – Contra) / (Ipsi + Contra). For the PHR test, the range of LI was between 0 (normal PHR, equal scores for the right and left sides of the body) and +1.0 (maximum ischemic deficit, PHR score for the right, contralateral side was 0). For the preference in the AT test, having in mind that the range of the preference was between 0% and 100%, the range of LI was from +1.0 (full ipsilateral preference or maximum ischemic deficit) through 0.0 (normal performance) to - 1.0 (full contralateral preference). For the latency in the AT test, having in mind that the range of the latency was between 1.0 s and (equal to the precision of measuring) 180.0 s, the range of LI was from approximately +1.00 (fast contralateral response and lack of ipsilateral response) through 0.00 (equal responses of both FLs, normal), to approximately −1.00 (fast ipsilateral response and lack of contralateral response, maximum ischemic deficit). The time sessions were T_0_, 1, 24, 72 h, where T_0_ was mean value of LIs of Preference or Latency before the Et-1 (or Saline) infusion on three consecutive days, with five trials on each day. The values of laterality index were normalized by subtracting the control values (at T_0_).

### Histological analysis

Mice and rats were sacrificed respectively at 48 and 72 h post MCAO and brains were fixed in Carnoy’s solution, embedded in paraffin, and sectioned at 10 μm. Sections regularly spaced every 550 μm (through the extension of the ischemic region) were deparaffinized and processed for staining with thionin (Nissl staining for histological assessment of neuronal degeneration). The infarct area was outlined at magnification of X 2.5 and measured with Scion Image software (NIH, Bethesda, MD, USA), then the infarct volume (V) was calculated by integrating the cross-sectional area of damage on each level and the distance between them: V = Σ (A_i_ x T_S__x n), where A_i_ is the ischemic area measured at i-th section, the T_S_ is the section thickness (10 μm), and n is the number of sections between two adjacent levels. In addition, we measured in rats the infarct area at level of a single section (AP = + 0.2 mm from bregma) in the S1FL, S1DZ, S1ULp, S2, GI/DI, AI and Cl.

### Statistics

For the neurological test in mice and the PHR test in rats, we used Kruskal-Wallis nonparametric ANOVA for multiple unrelated samples (Statistical package Statistica 7.0, 2004, Statsoft, Tulsa, OK) to determine the overall group effect at each time point. Then Mann-Whitney U-test (corrected for the small size of independent samples) was performed for the evaluation of the differences between Et-1 and sham-operated rats, separately for WT and *Grm2* mutant rats. Friedman ANOVA by ranks and subsequent Wilcoxon matched-pairs test for related samples were used for evaluation of changes in neurological scores in mice and asymmetry in the PHR test in rats as a function of time.

Statistical analysis of the grip strength test in mice and asymmetry in the AT test in rats was performed by three-way repeated measures Hotelling ANOVA (General Linear Model, Statistica7.0, Statsoft) with factors: Group (mGlu2^+/+^, mGlu2^−/−^), Side (Ipsilateral FL, Contralateral FL) and Time (T_0_, 2, 24 and 48 h) in mice, and two-way repeated measures Hotelling ANOVA for asymmetry in AT preference and latency with factors: - Group (WT, *Grm2* mutant) and Time (T_0_, 1, 24 and 72 h) in rats. For Post-hoc analysis we used Fisher’s LSD or Dunnett’s tests. Student’s’ t test was used for the analysis of infarct volumes in mice and rats (Statistica 7.0, Statsoft).
